# And yet... what makes the difference?

**DOI:** 10.1186/1471-2334-13-S1-P21

**Published:** 2013-12-16

**Authors:** Șerban Benea, Daniela Camburu, Mihaela Ionică, Manuela Podani, Otilia Elisabeta Benea

**Affiliations:** 1 National Institute for Infectious Diseases “Prof. Dr. Matei Balş”, Bucharest, Romania; 2 Carol Davila University of Medicine and Pharmacy, Bucharest, Romania

## Background

In the era of HAART *C neoformans* meningitis remains one of the most important opportunistic infections associated with HIV infection, with a high mortality (35-65%).

## Case report

We present 3 cases of *C neoformans* meningitis occurred in immunocompromised patients with advanced HIV infection (CD4<50 cells/cmm) caused by strains with susceptibility to fluconazole dose-dependent and different clinical course.

The first case: a 28 years old patient, confirmed with HIV infection in 2009. He is diagnosed with systemic infection with *C neoformans* with pneumonia, meningitis and cutaneous cryptococcosis and a CD4<50 cells/cmm. Blood and CSF cultures were positive for *C neoformans*. CSF changes were minimal, but with high pressure. Was treated with fluconazole - 1200 mg/day and lumbar punctures were performed repeatedly. CSF cultures were negative with difficulty, after about 8 weeks of treatment. The evolution was unfavorable with neurocognitive deterioration, seizures and death.

The second case: a 24 years old patient, diagnosed with HIV infection in childhood, with a history of multiple antiretroviral regimens but with discontinued treatment two years ago is diagnosed with *C neoformans* meningitis and a CD4<50 cells/cmm. CSF changes were minimal and CSF pressure was increased. Under treatment with liposomal amphotericin and lumbar punctures at 2-3 days intervals the evolution was slowly favorable. After 8 weeks of antifungal therapy the antiretroviral treatment has been resumed.

The third case: a 54 years old patient with confirmed HIV infection in 2011 is diagnosed with *C neoformans* meningitis and a CD4<50 cells/cmm. CSF had significant changes with incresed cellularity and low glycorrachia. Treated with fluconazole – 1200 mg/day plus flucytosine – the evolution was favorable.

## Conclusion

We discussed the different factors that determine the clinical course of *C neoformans* infection in HIV-infected patients with advanced immunosuppression.

